# Milk ladder as a therapeutic option for cow’s milk allergy: Proposal for a step-by-step plan for cow’s milk introduction in cow’s milk allergy

**DOI:** 10.5414/ALX02381E

**Published:** 2023-07-10

**Authors:** Amely Brückner, Petra Funk-Wentzel, Julia Kahle, Stephanie Hompes

**Affiliations:** 1 Elbe Klinikum Buxtehude, Clinic for Dermatology, Competence Center for Chronic Skin Diseases, Buxtehude,; 2 Practice for Nutrition Therapy, Stuttgart,; 3 Practice for Allergological Nutrition Therapy, Viersen, and; 4 Altona Children’s Hospital, Hamburg, Germany

**Keywords:** milk ladder, food ladder, step-by-step introduction of milk and milk products, non-IgE-mediated cow’s milk allergy, IgE-mediated cow’s milk allergy, baked milk products, acquisition of tolerance, patient selection

## Abstract

In regard to cow’s milk allergy, the current option of avoiding can be expanded by (re-)introducing milk using a milk ladder. So-called “food ladders” are internationally well known and utilized for both non-IgE-mediated and IgE-mediated cow’s milk allergy. Stepping up the stairs from highly processed baked goods with milk via cooked milk products to pasteurized fresh milk reflects the status of acquired tolerance of each level. The allergenicity of milk depends on processing and amount. By implementing the milk ladder, it can enhance the clinical process of tolerance development, lead to meeting nutrient requirements quickly, and involve parents actively in the therapeutical process. The milk ladder, for the first time being published and adapted for Germany, describes a structured framework that might be adapted individually regarding the time period on a certain level or other variations such as preparation/amount of milk products. From a safety perspective, healthcare professionals should pay great attention to patient selection and education prior to implementing the milk ladder. Detailed advice as well as recipes and a graphical presentation can be found in the supplemental material.

## Introduction 

Stepwise food (re-)introduction plans are widely used internationally as part of the therapeutical management of food allergies [1]. Most food ladders involve the staple foods milk and egg. The typical indication for the stepwise plan for cow’s milk introduction is non-IgE-mediated cow’s milk protein allergy, but it is also increasingly used internationally for IgE-mediated cow’s milk allergy [2]. The current strategy for treating cow’s milk allergy is to avoid milk and dairy products [3]. 

Data from the EuroPrevall study show a 2-year incidence of cow’s milk allergy of 0.54% in infants aged 1 – 2 years. 23.6% had non-IgE-mediated cow’s milk allergy. From this group, all children were tolerant after 1 year, and among IgE-mediated cow’s milk allergy sufferers, 57% were tolerant [4]. The underlying immunological processes of tolerance development in IgE-mediated food allergies are based on a series of changes in the cross-linked immune responses of T and B lymphocytes and their interleukins. The development of tolerance in non-IgE-mediated food allergies has not been clearly elucidated^5^. However, it has been shown that tolerance occurs significantly earlier in non-IgE-mediated compared to IgE-mediated allergies [6]. In contrast to strict avoidance, introduction of baked cow’s milk products may also accelerate tolerance to unheated dairy products [7, 8]. 

Testing for acquired tolerance to cow’s milk-containing foods can be performed either by oral food challenges in a clinical setting or, for selected patients, by using the “milk ladder”. The milk ladder can be used to successively check the degree of existing tolerance in a home setting [9]. 

As the implementation of the milk ladder is not free of risks, certain criteria such as young age (up to preschool), no risk of anaphylaxis, no existing or well-controlled asthma, and adherence should be considered for patient selection [10]. FPIES (food protein-induced enterocolitis syndrome) and EoE (eosinophilic esophagitis) are not indications for a milk ladder. So-called food ladders should be distinguished from oral immunotherapy (OIT). In contrast to food ladders, OIT is a therapeutic option for persistent food allergy. Initiation of therapy and any increase in allergen dose must be carried out under medical supervision. At home, the allergen must be given daily [11]. So far, OIT is only approved for peanut [12]. The therapeutic goal is to raise the threshold dose so that allergic symptoms no longer occur when the smallest amounts are inadvertently consumed [13]. In contrast, with food ladders, ideally complete tolerance should be achieved at the end of the application. 

## History and further development of the milk ladder 

The first “milk ladder” was part of the 2013 Milk allergy in Primary Care (MAP) Guidelines with a 12-step plan based on UK diets [14]. This form of cow’s milk introduction was originally reserved for mild to moderate forms of non-IgE mediated cow’s milk allergy (excluding FPIES and EoE) [14]. A further development for international use was published in 2017 with a shortened milk ladder version over 6 stages (iMAP) [2]. Recipes for different stages with precise information on milk protein contents as well as baking or cooking temperatures have been published [2]. These, or modified forms of the milk ladder, are now widely used internationally and in some cases include recipes with local preparation differences [7, 15, 16, 17, 18, 19]. In addition, the milk ladder is now used not only for non-IgE-mediated but also for IgE-mediated cow’s milk allergy [20]. 

## Milk ladder – goals and management 

The aim of a gradual introduction of milk is to determine the already existing tolerance threshold as well as to support the further course of tolerance development and to limit food avoidance to a (temporal) minimum, also with regard to ensuring the nutrient supply. In this respect, the milk ladder enables an increase in food variety and food diversity. Through structured introduction of milk-containing foods, parents of the affected children regard themselves actively involved in the therapeutical process [21, 22]. With increasing quantity and less processing, the overall nutrient supply and especially the intake of protein and calcium improves. 

In addition, the long waiting time for an inpatient food challenge can be circumvented, thus enabling a significantly earlier introduction of milk products in many cases. In addition, parents who are critical of inpatient oral food challenge and whose children otherwise run the risk of continuing milk avoidance for an unnecessarily long or even indefinite period are reached in this way [1]. 

An important prerequisite for its use is that parents have a good understanding of the implementation and principle of the milk ladder and can put it into practice. Otherwise, it carries the risk of allergic reactions, for example, by advancing too quickly on the ladder or by the premature consumption of products that are not well baked [13, 23]. In addition, in the authors’ experience, the risk of an allergic reaction is particularly increased during the transition from long-baked to rather short-heated products, e.g., from muffin to pancake. 

## Starting challenge under medical supervision 

There is the possibility to precede cow’s milk challenge with a single administration of a very small amount of milk. In the working group of J. Hourihane from Ireland, this single-dose challenge was made the starting point of a milk ladder. Here, only 1 drop of milk was administered to the cow’s milk-allergic infant immediately after diagnosis for testing purposes [24]. This corresponds to the amount of ~ 0.015 mL of fresh cow’s milk or 0.5 mg of cow’s milk protein, the amount internationally referred to as the threshold dose for objectifiable symptoms in 5% of cow’s milk-allergic patients (ED_05_) [25]. On the one hand, this allows high-risk patients to be filtered out, and on the other hand, it showed that the group with the single-dose challenge progressed faster and finished earlier on the milk ladder than the group without the preceding food challenge. The authors attribute this to the fact that this single-dose administration reduced anxiety in the parents [24]. 

## Composition, processing, and allergenicity of cow’s milk and dairy products 

Numerous allergens have been detected in cow’s milk, of which casein and β-lactoglobulin are particularly relevant. Cow’s milk consists of 80% various caseins and 20% whey proteins [26]. 

Baking, cooking, frying, but also fermentation change the allergenicity of foods. Exposure to heat leads to denaturation of the conformational epitopes, while linear epitopes tend to remain intact. The individual protein fractions in cow’s milk react differently to heat: casein is widely heat stable, whereas α-lactalbumin and β-lactoglobulin are heat labile [23]. Conformational epitopes of β-lactoglobulin can be reduced by 99% from 680 µg in a muffin batter to 0.17 µg in a baked muffin [26]. Casein remains stable during baking even after 60 minutes. β-lactoglobulin is undetectable after ~ 15 – 20 minutes [27]. Milk baked into wheat matrix reduces the binding ability for antibodies [28]. These denaturation processes have an impact on compatibility depending on the sensitization pattern. 

Thus, a large proportion of children with IgE-mediated cow’s milk allergy tolerate baked dairy products over time, but also other processed forms of cow’s milk such as hard cheese [29]. On the contrary, in children who cannot tolerate baked products containing cow’s milk, the degree of heating or processing of cow’s milk does not seem to affect the reaction threshold [30]. 

## Time regimen – dwell time and increase 

With regard to the duration of staying on a step of the milk ladder, there are no strictly defined timeframes. If a child tolerates a step safely, i.e., has no complaints in various everyday situations, it is possible to move on to the next ladder step. 

It is recommended to consume small amounts of the next stage first and then slowly increase [20]. The Canadian Milk Ladder recommends starting with a crumb or pea-sized amount of baked milk. The food should be given daily and the amount gradually increased to an age-appropriate amount. It is recommended to remain at one level for at least 1 – 3 months before moving to the next level [17]. However, this makes the process very lengthy. 

Other authors recommend progressing from one step to the next after the child has tolerated 3 cow’s milk meals per week in age-appropriate amounts without symptoms [24]. This approach seems very practicable and allows relatively rapid progression up the milk ladder. 

If an infection with fever occurs, climbing another ladder step is not recommended. Rather, the child should then take a short break and initially remain on the same step for a few days. If symptoms occur in a clear temporal connection with the consumption of the food containing milk, the amount or preparation previously tolerated should be reduced. In addition, other co-factors such as physical exertion may play a role. 

The milk ladder does not necessarily have to be started with step 1 if the dietary history provides evidence of products that are already tolerated. Depending on tolerability, it can be started at a higher step. In the same way, a successful challenge test with baked milk can be used as a starting point for the milk ladder. 

## Safety through careful patient selection 

After the fatal anaphylaxis in a 9-year-old Canadian girl with uncontrolled asthma and persistent IgE-mediated cow’s milk allergy on May 20, 2021, after consumption of a small amount of baked milk in the context of the milk ladder, this and oral immunotherapies with milk are under discussion once again. Professional societies call for a uniform procedure and criteria defining in which patients the milk ladder can be used safely [1]. 

Most food ladder studies included only preschool-aged patients who met the following conditions: no history of anaphylaxis, no existing asthma or well-controlled asthma, and a family history of good self-management. Or the patients had already had a successful food challenge in which baked milk was tolerated [31]. 

According to the British professional society BSACI, only patients without the risk of anaphylaxis, who are under 5 years of age and are very likely to grow out the milk allergy, should be scheduled for a milk ladder. For older patients in whom natural tolerance development is highly unlikely, use of the milk ladder is not recommended. In these cases, structured oral immunotherapy with cow’s milk would be necessary, but this carries significantly higher risks of allergic reactions [32]. 

Patients intended for the milk ladder or their parents should be informed in detail about the procedure, possible risks, and benefits. It is advisable to document the implementation of the individual steps of the step-by-step plan. In case of questions or problems, contact with the doctor and/or nutritionist should be possible, and selected control appointments should be made. 

With regard to patient selection, the following criteria should be considered (modified according to BSACI and the Canadian Society of Allergy and Immunology: Joint Statement on oral immunotherapy, BSACI website, 2022) [31]:

- The age of the patients should not exceed preschool age. The younger the better!
- Indications of possible tolerance from the exact nutritional history can be picked up and taken into account (e.g., tolerance of baked milk and thus onset at higher ladder level quite possible).
- No asthma is ideal; existing asthma should be well controlled.
- No patients with anaphylaxis with respiratory or cardiovascular symptoms in temporal relation to cow’s milk consumption.
- A low threshold dose should not have caused any reaction.
- Parents should be able to recognize emergency situations, administer emergency medications as needed, and get help quickly.
- The patient, and therefore the parents as caregivers, must be adherent.
- Parents must be able to recognize when the next higher step is possible, should be suspended for a short period of time, or should be delayed.
- Atopic eczema and other underlying diseases should be well controlled.
- There should not be any communication or language barriers between families and treatment providers.

## Conclusion 

After careful patient selection, taking into account the above criteria, the implementation of the milk ladder may be an appropriate method for cow´s milk introduction. 

In the Supplemental Material (Appendices 1 – 3), the 6-step milk ladder for introducing cow’s milk products is presented with recipes for practical application. The procedure according to the milk ladder represents a framework. Deviations such as a faster or slower procedure and smaller or larger consumption quantities can be individually adapted. To ensure good tolerance, make sure that baked goods in particular are well baked. In principle, a balanced, healthy diet in line with requirements is recommended. 

## Funding 

None. 

## Conflict of interest 

The authors declare that there is no conflict of interest. 

## Supplemental material

Supplemental material Appendices 1 - 3
