# Supplementary material for: Milk ladder as a therapeutic option for cow’s milk allergy: Proposal for a step-by-step plan for cow’s milk introduction in cow’s milk allergy
Source: Allergol Select. 2023 Jul 10;7:116–21. doi: 10.5414/ALX02381E (PMC10339713; doi:10.5414/ALX02381E)
Supplement: Supplemental material [file allergologieselect-7-116-S01.pdf]

Appendix 1: Milk ladder education for parents/caregivers of cow's milk-allergic children

(modified from The iMAP Milk Ladder – an international interpretation of the MAP (Milk Allergy in Primary Care) guideline, 2017 [2] and Thomas et al. Use of an egg ladder for home egg introduction in children with IgE-mediated egg allergy. Pediatr Allergy Immunol. 2021; 32: 1572-1574) [33].

Milk ladder/step by step plan for the introduction of cow's milk in the case of cow's milk allergy

[Created 4/2023 by Amely Brückner, Petra Funk-Wentzel, Julia Kahle, Stephanie Hompes]

You should only implement this step-by-step plan for introducing cow's milk to your child upon recommendation and in consultation with your treating pediatrician/allergist. The plan can only be used in children who have had a mild to moderate reaction to cow's milk. It should not be carried out in children with severe allergic reaction (anaphylaxis) to cow's milk.

Cow's milk is found in many foods in a wide variety of forms. Heating, processing, or baking cow's milk causes a structural change in the protein. As a result, a large proportion of children can tolerate baked dairy products such as muffins and cookies before they can drink less heated or pure milk.

Notes on the application of the step-by-step plan:

- Before starting the introduction or moving to the next level, you should always be sure that your child is well and that there is no illness.
- Start with level 1 (cookies). If your child already eats cookies, start with the next level.
- When starting the step plan or moving to the next step, please make sure you have your child under observation for at least 2 hours after eating the new food.
- Always move to the next level only after your child has eaten the food from the current level with the largest amount listed several times without symptoms. The speed of increase can vary greatly from child to child.
- Once milk from one stage has been successfully introduced, that food, e.g., muffin, should continue to be eaten regularly while the product from the next stage, e.g., pancakes, is (re-)introduced.

What to do if an allergic reaction occurs?

Emergency medications should be used according to the plan (as discussed with physician). Thereafter, medical stuff should be contacted to discuss how to proceed. The dairy foods that have been well tolerated previously should continue to be given regularly. Your child may need a longer period of regular consumption before moving to the next stage.

Appendix 2: Graphical presentation of the milk ladder with information on the stages (food, processing, proportion of cow’s milk/protein per food)

Milk ladder = step-by-step plan for cow’s milk introduction				Average protein content
[Created 4/2023 by Amely Brückner, Petra Funk-Wentzel, Julia Kahle, Stephanie Hompes]				
– Individual **allergological assessment** necessary **before starting**!<br>– Start with step 1 and move to next step in case of tolerance.<br>– For step 1 to 4, the proven recipes can be used.<br>– **Give the food of the milk ladder regularly** (several times a week)!<br>– If symptoms occur with a larger amount or new levels, go back one step and try to increase again after a while.				
Step 6	**Milk**<br>= fresh milk pasteurized or infant formula	100 – 200 mL pasteurized milk		3.5 – 7.0 g
Step 5	**Yoghurt, cheese**<br>fermented dairy products	15 – 30 g cheese<br>30 – 60 g cream cheese<br>60 – 125 g natural yoghurt****		3.7 – 7.4 g<br>3.3 – 6.6 g<br>2.3 – 4.8 g
Step 4	**Pizza, rice pudding, cheese**<br>strongly heated, milk long boiled	7 – 15 g cheese on pizza/casserole<br><br>1 serving rice pudding**<br>1/2 portion rice pudding**	contains milk in mL:<br><br>100<br>50	1.8 – 3.7 g<br><br>3.5 g<br>1.7 g
Step 3	**Pancake**<br>larger quantities of milk, baked with flour, butter***	1 pancake**<br>1/2 pancake**	42<br>21	1.5 g<br>0.7 g
Step 2	**Muffin**<br>milk baked with flour	1.5 muffins**<br>1 muffin**<br>1/2 muffin**<br>1/4 muffin**	37.5<br>25.0<br>12.5<br>6.25	1.3 g<br>0.9 g<br>0.4 g<br>0.2 g
Step 1<br>Start	**Pastry, rusk**<br>smallest amounts of milk, baked with flour	3 cookies**<br>2 cookies**<br>4 wheat rusks* or 1 cookie**<br>2 wheat rusks* or 1/2 cookie**<br>1 wheat rusk* or 1/4 cookie**	3.0<br>2.0<br>1.0<br>0.5<br>0.25	0.1 g<br>69 mg<br>35 mg<br>17 mg<br>8 mg

*Calculated for Brandt Zwieback (wheat rusk), do not use wholemeal rusk or other brands, these deviate in the amount of milk! 1 “Brandt Zwieback”, ~ 9.4 g, corresponds to ~ 3 Mini-Zwieback from Brandt.
**Use proven and calculated recipes.
***Butter additionally possible as spreadable fat. 5 g butter (1 tsp) = 1.75 mg milk protein.
****100 g natural yoghurt “stichfest” (semi-solid) contains ~ 3.8 g protein/100 g.

Appendix 3: Recipes/preparations for steps 1 – 4 of the milk ladder

[Created 04/2023 Amely Brückner, Petra Funk-Wentzel, Julia Kahle, Stephanie Hompes]

Recipes

Step 1: Cookies
(About 20 finger-sized cookies. 1 cookie contains 1 mL of milk).

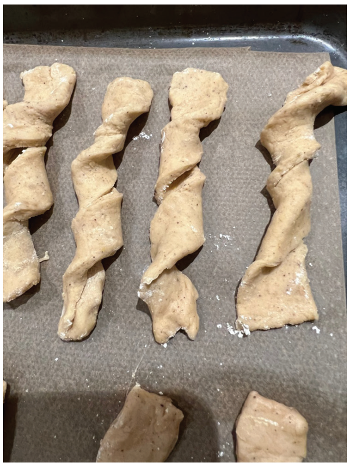

Figure 1. Cookie dough with sweetener banana and milk powder (Image copyright: A. Brückner).

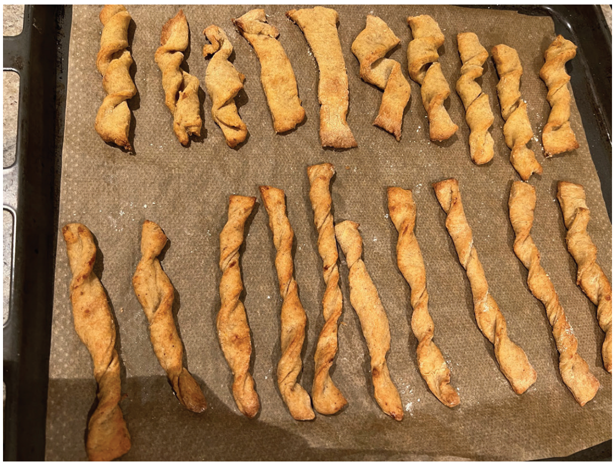

Figure 2. Finger-length cookies with milk powder (Image copyright: A. Brückner).

Ingredients:
- 125 g flour (replace with gluten-free flour mix if needed).
- 2 g skim milk powder (with 32 – 35 g protein/100 g, e. g. Sucofin or Saliter skim milk powder)
- 50 g margarine (non-dairy)
- 1/4 – 1/3 cup mashed ripe banana (or 30 g sugar)
- pinch of vanilla sugar

Preparation:
1. Preheat convection oven to 180°C (if using top/bottom heat 200°C).
2. Mix flour and skim milk powder.
3. Add margarine and mashed banana (or sugar) and stir in.
4. Let dough rest in refrigerator for ~ 30 minutes.
5. Roll out the dough into a rectangle on a floured surface and cut into finger-width strips.
6. Bake for ~ 15 minutes.

Tip:
When using banana, it is recommended to increase the baking time by a few minutes.

Notice:
Skim milk powder enables an even distribution of the milk protein.

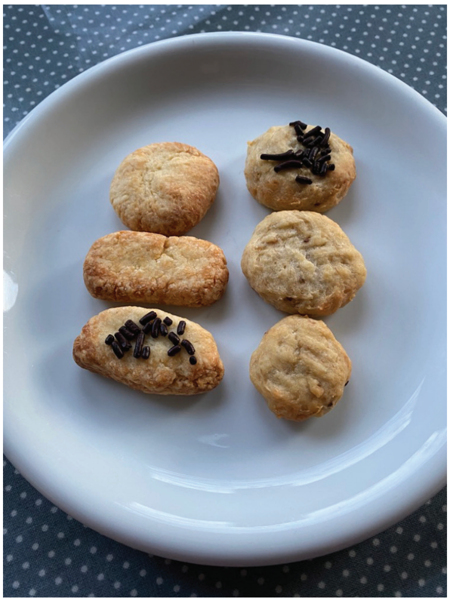

Figure 3. Wheat flour cookies containing milk powder with sweetener banana and optionally with plain sprinkles (Image copyright: S. Hompes).

Step 2: Muffins

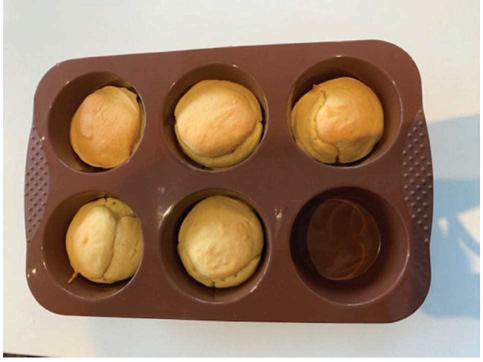

Figure 4. Muffins with milk and applesauce as sweetener (Image copyright: J. Kahles).

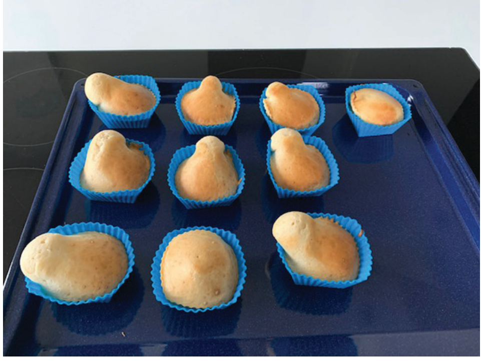

Figure 5. Gluten-free dairy muffins with applesauce as sweetener (Image copyright: J. Kahles).

Ingredients for sweet muffins: (10 pieces; 1 muffin contains 25 mL of milk)
- 250 g wheat flour, type 405 (replace with gluten-free flour mix if needed)
- 10 g baking powder
- 25 g sugar
- 1 pinch salt
- 50 mL neutral oil, e.g., rapeseed oil
- 250 mL fresh milk
- 80 g applesauce (alternatively 110 g mashed banana or 110 g chunky fruit such as apples, apricots, blueberries)
- (for gluten-free flour: use 120 g applesauce or 160 g fruit).
- If necessary, refine with cinnamon, grated untreated lemon or vanilla (sugar)

Ingredients for savory muffins: (10 pieces; 1 muffin contains 25 mL of milk)
- 250 g wheat flour, type 405 (replace with gluten-free flour mix if needed)
- 10 g baking powder
- 1 pinch salt
- 50 mL neutral oil, e.g., rapeseed oil
- 250 mL fresh milk
- 40 g cubed bacon
- 40 g vegetables (small pieces of bell pepper, zucchini, corn, etc. – depending on preference)

Preparation:
1. Preheat convection oven to 180 °C (if using top/bottom heat 200 °C).
2. Mix flour, baking powder, (sugar), and salt.
3. Beat oil and milk with mixer until frothy and add to dry ingredients.
4. Add chopped fruit (for sweet muffins) or cubed bacon with vegetables (for savory muffins) and fold in.
5. Divide batter evenly among 10 muffin tins and bake for 20 – 25 minutes.

Important:
Use a toothpick to check that the muffins have been baked long enough. No more batter should stick to the toothpick. Baking time may vary from oven to oven.

Tips:
- If tolerated, add 1 egg in addition to the above ingredients, especially if using gluten-free flour.
- The muffins are easier to release from a silicone baking pan.
- The muffins are easy to freeze and thaw. If necessary, you can cut the muffins in half after thawing, toast them and top/coat them.

Step 3: Pancakes

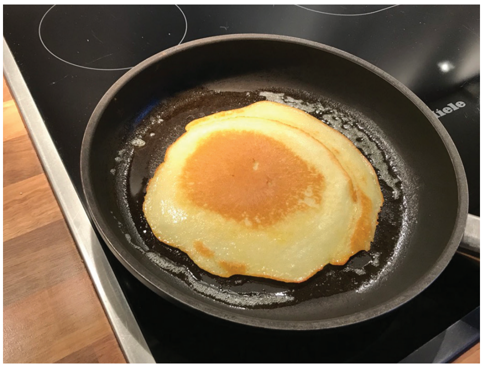

Figure 6. Gluten-free pancake with milk and chicken egg (Image copyright: S. Hompes).

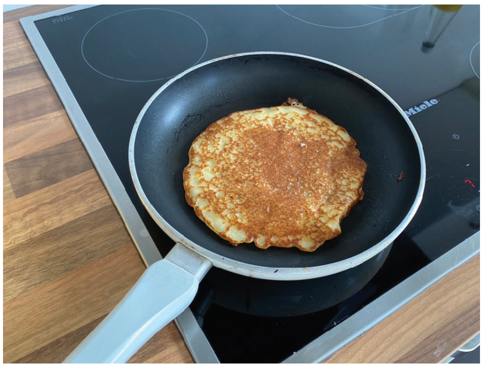

Figure 7. Gluten-free pancake with milk without chicken egg (Image copyright: S. Hompes).

<u>Ingredients for 6 pieces:</u> (1 pancake contains 42 mL of milk.)

- 125 g wheat flour, type 405 (replace with gluten-free flour mix if needed)
- 1 pinch baking powder
- 1 pinch salt
- 250 mL fresh milk
- 1 egg (in case of egg allergy, replace with 50 mL water plus 30 mL oil, e.g., rapeseed oil, or use egg substitute).
- Oil for frying

<u>Preparation:</u>

1. Whisk the egg (or egg substitute) with oil and salt in a bowl and gradually stir in the flour with the baking powder and the milk to make a smooth dough.
2. Over medium heat, cook 6 pancakes of equal size until golden brown on each side. (To get 6 pancakes of equal size, it may be helpful to divide the batter into 6 cups first and then bake those portions in the pan).

Attention! The dough without egg burns more easily, so reduce the heat a little in this case.

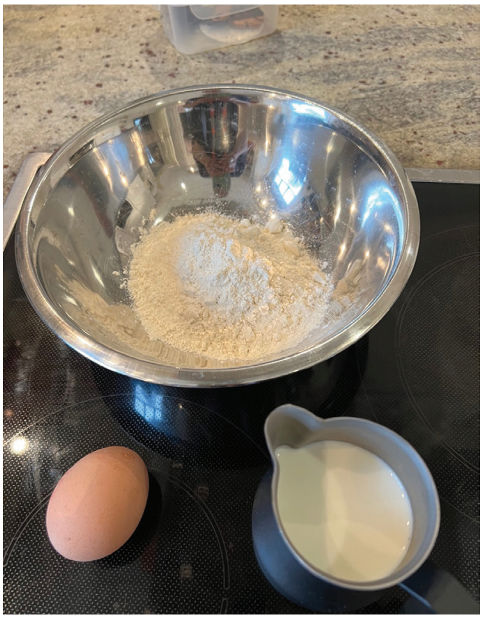

Figure 8. Wheat pancakes with milk and hen's egg (Image copyright: A. Brückner).

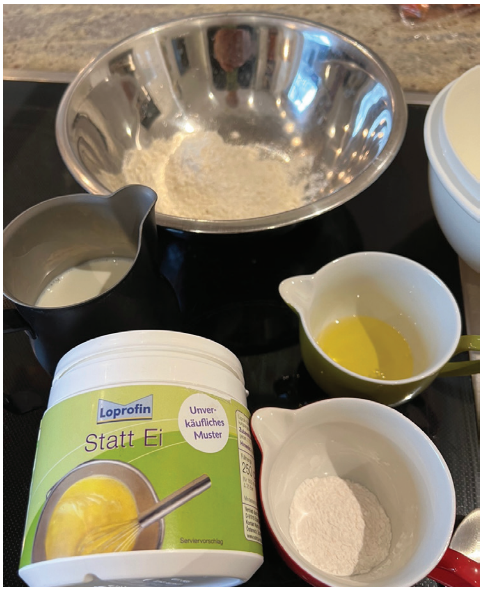

Figure 9. Wheat pancakes with milk and egg substitute instead of egg from Loprofin (egg substitute based on potato starch) (Image copyright: A. Brückner).

Step 4: Rice pudding

Ingredients for one serving: (1 serving contains 100 mL of milk.)

- 100 mL milk
- 25 g rice pudding

Preparation:

1. Bring the milk to the boil briefly in a saucepan and then add the rice pudding, stir briefly.
2. Turn the stove temperature down so that the milk is still simmering slightly but no longer boiling. Cook the rice pudding, stirring several times, for about 30 – 35 minutes until the desired consistency is reached.

Tips:

- Can be eaten cold or hot.
- Serve with cinnamon and sugar, jam, or compote.

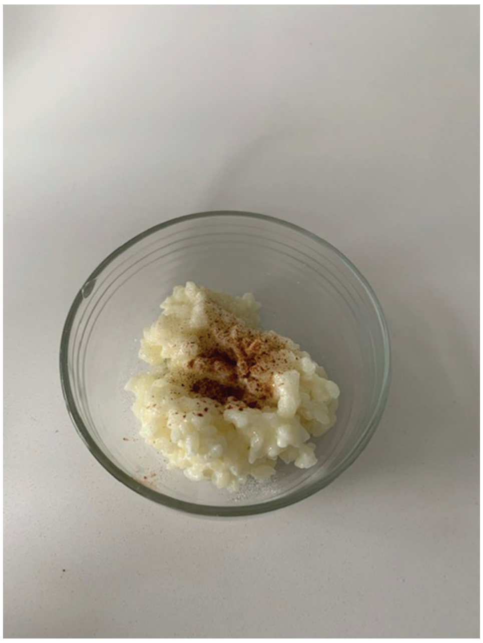

Figure 10. Rice pudding (Image copyright: J. Kahle).
